# Effects of an Ozonated Water Irrigator on the Plaque Index and Bleeding Index of Pregnant Women

**DOI:** 10.3390/jcm11144107

**Published:** 2022-07-15

**Authors:** Simona Tecco, Alessandro Nota, Teresa D’Amicantonio, Laura Pittari, Marika Monti, Elisabetta Polizzi

**Affiliations:** Dental School, Vita-Salute San Raffaele Hospital and I.R.C.C.S. San Raffaele Hospital, 20132 Milan, Italy; nota.alessandro@hsr.it (A.N.); damicantonio.teresa@hsr.it (T.D.); laura_pittari@hotmail.it (L.P.); monti.marika@studenti.unisr.it (M.M.); polizzi.elisabetta@hsr.it (E.P.)

**Keywords:** pregnant women, gingivitis, bleeding on probing, gingival, ozone, ozonated water, dental plaque

## Abstract

Pregnancy causes physiological changes in the woman’s body, which can also affect oral health. Therefore, pregnant women may manifest gingival inflammation that is favored by their hormonal increase. This clinical trial (ClinicalTrials.gov Identifier: NCT04140643) evaluated the variation in the plaque index (PI) or bleeding index (BOP) in pregnant women after daily use of an ozonated water irrigator and proper home oral hygiene, compared to a control group who only performed home oral hygiene. The inclusion criteria were the gestation period from the 14th week to the 30th week, a diagnosis of gingivitis, and a minimum number of teeth equal to 20. The PI and BOP index were evaluated at T0, fifteen days after T0 (T1), and two months after T1 (T2). The PI values systematically decreased over time (F (1.19) = 41.82) in both groups, with a systematic difference in PI values between the two groups (F (1.19) = 6.28, *p* = 0.021). A statistically significant difference was assessed in the BOP index at T2 between the two groups, with the control group suffering a higher BOP index. The results show that the patients in the study group showed a decrease in the BOP index over the three time points, in contrast to the control group, due to the beneficial properties of ozonated water.

## 1. Introduction

Pregnancy is characterized by complex physiological changes in the woman’s body, which can also affect oral health. As numerous physiological and pathological conditions, including obesity and metabolic syndrome, are related to microbial changes, called dysbiosis, it is therefore not surprising that there are also significant changes in the microbiota during pregnancy, when dramatic weight gains and metabolic and immunological changes occur [1]. During pregnancy, the composition of the subgingival microbiome is shifted toward complexes associated with periodontal disease [2]. In addition, changes in the microbial composition during pregnancy affect a variety of sites in the body, including the intestines, the vagina, the oral cavity, and the placenta, and are vital for a healthy pregnancy [3]. Microbial changes are likely coordinated with immune, endocrine, and metabolic states [1]. Hormonal, vascular, and immunological modifications can generate an exaggerated inflammatory response in the gingival tissues against pathogenic microbes of the oral biofilm. Therefore, the pregnant woman may manifest gingival inflammation favored by the hormonal increase that causes an increase in vascularization and capillary permeability, and a decrease in immune defenses, predisposing the gingival tissues to the development of inflammation. Moreover, periodontal tissue inflammation, due to biofilm formation, increases dramatically in size and severity during a normal pregnancy, even without changes in the amount of biofilm present. From a clinical point of view, intraoral disorders, which occur most often during the gestation period, are favored by hormonal changes, often combined with a lack of routine intraoral examinations and delays in the treatment of oral diseases [4]. Among the most frequently cited diseases in the literature are pyogenic granuloma, gingivitis, and periodontitis. In addition, a decrease in salivary pH is observed in pregnant women which can lead to a higher incidence of dental caries in this period [1]. Finally, changes in diet and oral hygiene, morning hyperemesis gravidarum, and gastroesophageal reflux disease can cause the demineralization of the dental tissues with erosion of the enamel and an increased risk of caries. Periodontal pathogenic bacteria represent a possible source of infection in pregnant women by the fact that they can enter the bloodstream, leading to some unfavorable outcomes such as an underweight birth or a pre-term birth [2]. Changes in the oral microbiome of pregnant women and possible oral consequences were also registered in patients with COVID-19, mostly concerning those bacteria that use progesterone as a source of nutrition and are implicated in the development of periodontal disease [5].

Improving oral hygiene practices has been demonstrated to have the potential to maintain the oral micro-ecological balance [6]. The pregnant woman is therefore considered a special-needs patient, as she needs special and personalized attention to the oral problems that may arise during the gestational period. In the dental field, anti-inflammatory drugs are administered to treat typical gingival inflammation. As reported by the Italian Ministry of Health, the safe use of substances and drugs during pregnancy is related to various factors including the time of administration in pregnant patients, which is considered a factor that should be kept under control to reduce the risk of complications for the fetus and the mother [7]. Chlorhexidine or alcohol-free mouthwash should therefore be used during pregnancy only when the clinical need has been clearly highlighted by the dentist [8,9].

For this purpose, given the lack of specific aids that can prevent and/or treat the state of gingival inflammation typical of the gestational period, without having negative effects on the fetus, our scientific institute, I.R.C.C.S. San Raffaele Hospital, proposed a monocentric study aimed to study an ozonated water irrigator called Aquolab© (Aquolab© EB2C, Milano, Italy) in the prevention/treatment of gingival inflammation.

Ozonated water and ozone have a disinfectant power, reduce the signs of inflammation, and inhibit the formation of bacteria that cause halitosis, as demonstrated by the various articles published in the literature on the use of ozone therapy in the dental field [10,11,12,13]. They were demonstrated to be efficacious in the management of chronic periodontitis [14,15] and oral lichen planus [16], without the toxic effects of dental pulp cells [17]. Overall, other recent data suggest that ozonated water suppresses C. albicans growth and biofilm formation on polymethyl methacrylate (a dental prosthetic material) without impairing surface properties [18]. It was also related to a beneficial role in the management of peri-implant mucositis sites [19]. Ozonated water does not create any problems either for the pregnant woman or the fetus, as reported in the toxicity report from the manufacturer; therefore, based on what was previously evaluated, it can be stated that the use of Aquolab© does not involve any health hazard with reference to the potential dispersion of ozone in the environment and consequent toxicity to exposed persons [20].

Thus, the aim of the present study was to evaluate any variation in the plaque index or bleeding index in pregnant women after daily use of Aquolab© and proper home oral hygiene, compared to a control group who used only home oral hygiene.

## 2. Materials and Methods

The present randomized clinical trial was performed on a total sample size of 30 women; after a simple randomization procedure performed using sealed envelopes, 18 subjects (mean age 37.6 ± 2.8) were included in the study group and 12 subjects (mean age 35.2 ± 4.8) in the control group.

**Figure 1:**
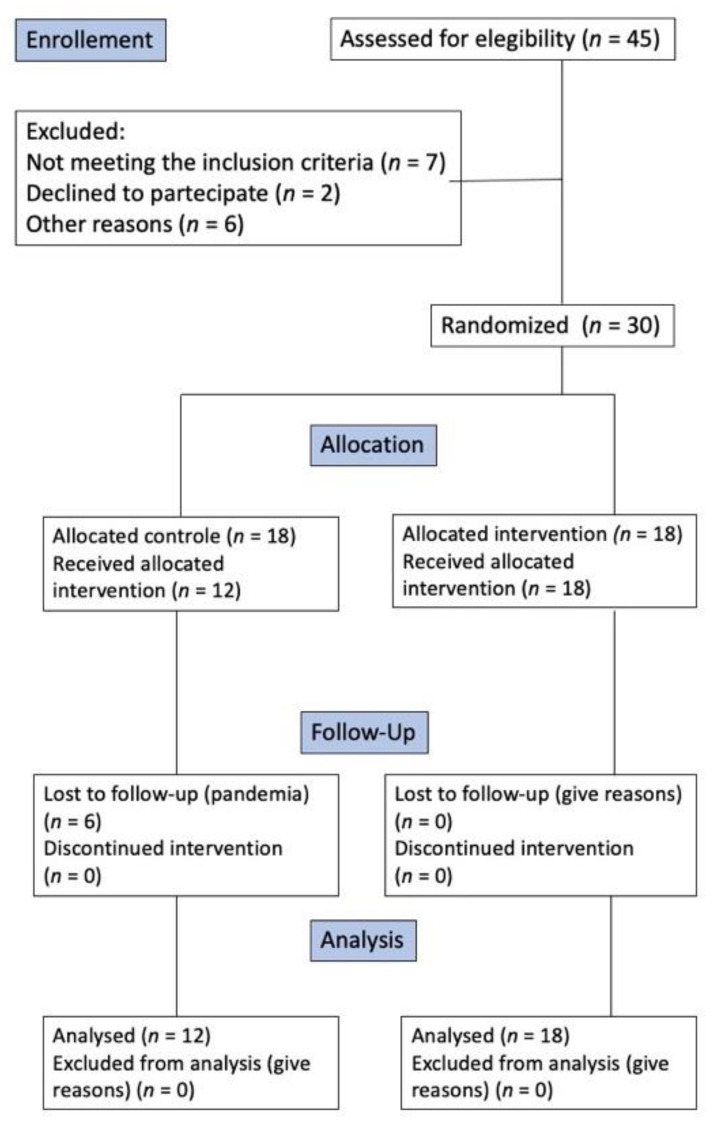
CONSORT flow chart of the study.

The protocol was approved by the Ethics Committee of the San Raffaele Hospital with the EB2C document on 11 October 2018. Pregnant women who had the following inclusion criteria were recruited from the San Raffaele Hospital: gestation period from 14th week to 30th week, diagnosis of gingivitis, and minimum number of teeth equal to 20. Moreover, the following exclusion criteria were adopted: consumption of alcohol and/or smoking, systemic diseases, drugs, allergies, orthodontic appliances, and a positive periodontal screening and recording index (PSR) [21,22,23]. The recruitment was carried out with the support of the Gynecology and Obstetrics Operating Unit which gave the opportunity to organize a weekly meeting dedicated to the presentation of the project. At the end of the meeting, the pregnant women who were interested could schedule their first appointment of the project. Clinical procedures were conducted by two operators identified below as operator A and operator B who attended the visits of the patients during the study (at T0, T1, and T2). These operators are authors of the study (TDA and MM). As represented in Figure 1, the timeline of the study was as follows: at T0, each patient, after completing a proper medical history questionnaire and informed consent, underwent the first dental visit and a survey of clinical parameters, such as the plaque index (PI) and bleeding on probing index (BOP); then, a professional oral hygiene session was performed by operator A.

At the end of the session, operator B randomly placed each woman in one of the following groups: group 1 (the study group), which included subjects who received home oral hygiene instructions and an ozonated water irrigator device, with the appropriate instructions for its use; or group 2 (the control group), which included women who received only home oral hygiene instructions without the machine. The home oral hygiene instructions were as follows: (a) use a manual toothbrush with soft bristles or an electric brush with an ultrathin head twice a day for 4 min; (b) use interdental aids (dental floss or a brush, once a day); (c) use a fluorinated toothpaste as a remineralizer for the hard dental tissues; (d) use Aquolab© once a day according to the instructions provided by the manufacturer: ozone level 2, water level 1, with the 0.8 mm nozzle, air pump of 75% PWM with an irrigation time-out of 100 s, and tension of 12 V (these were given only to the women included in the study group).

At T1, 15 days after T0, a re-evaluation of the clinical parameters was performed by operator A. At T2, two months after T1, a new re-evaluation of the clinical parameters was performed by operator A. Operator A was blinded to the group of the patient.

**Figure 2:**
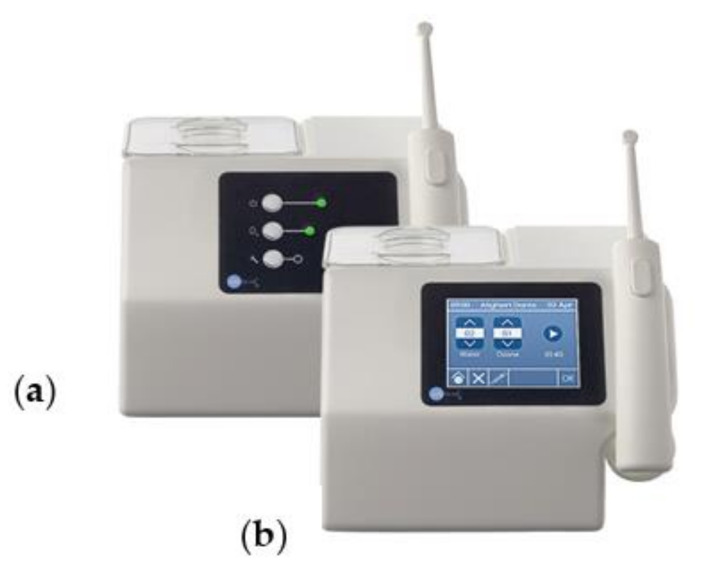
The Aquolab© devices: (**a**) home device; (**b**) professional device.

The ozonated water irrigator (Aquolab© EB2C, Milano, Italy) used for this study is shown in Figure 2. This water irrigator is different from common water jets: its effectiveness is not based on the power of the action of water, but rather on the action of ozone mixed with water. Oxygen–ozone therapy is a mild technique that exploits the potentials of ozone combined with oxygen to stimulate and increase the protection mechanisms against the production of free radicals, with a consequent reduction in substances that are toxic for the cells [17].

### 2.1. Sample Size

A sample size calculation was carried out on a preliminary sample of 20 subjects, showing that to achieve a minimum power of 80%, with an alpha error of 5%, a minimum number of 12 subjects per group was necessary. Thus, the enrollment of the subjects was carried out with the simple randomization procedure until a minimum of 12 subjects per group was obtained, after which another 6 women participated and were included in the study group.

### 2.2. Data Handling and Statistical Analyses

The statistical analysis of the data was carried out through the values of the PI and BOP index, at T0, T1, and T2. The descriptive statistics and inferential tests were calculated setting the *p* value at 0.05%. The descriptive statistics included the mean and the standard deviation. Between-group differences were calculated using Student’s *t* test for different samples. Intra-group differences over time were screened with the repeated measures analysis of variance. 

## 3. Results

Table 1 shows data of the PI.

The patients belonging to the study group started at T0 with a lower PI than the patients belonging to the control group, who started with a higher PI. However, the repeated measures analysis of variance found (a) that the PI values systematically decreased over time (F (1.19) = 41.82) in both groups, and (b) that a systematic difference was observed in the PI values between the two groups (F (1.19) = 6.28, *p* = 0.021). Table 2 shows the BOP index over time in the two groups.

The repeated measures analysis of variance found that the BOP values systematically decreased over time in the study group. The trend of the BOP index suggests an improvement in the treated group, as a statistically significant difference was assessed at T2 between the two groups, with the control group suffering a higher BOP index. 

## 4. Discussion

From the results obtained from the statistical analyses, it can be observed that the PI decreased over the three time points both in the treated group and in the control group. This result is justified by the fact that all the patients in the two groups were motivated through repeated home oral hygiene instructions given by dental hygienists. All the included subjects were instructed to use interdental aids and a toothbrush correctly and were foreseen at each visit for a personalized brushing technique, therefore being motivated to perform correct home oral hygiene. Therefore, the results suggest that pregnant women who have been re-motivated and educated to perform correct home oral hygiene show an improvement in their PI over time. 

The analyses performed on the BOP index revealed that the subjects in the study group showed a decrease in their BOP values over the three time points, in contrast to the control group, which suffered a partial increase between T1 and T2. From the results obtained on the BOP index, it seems that subjects in the study group, compared to the patients in the control group, had a decrease in the BOP index over time, due to the beneficial properties of ozonated water.

Nearly 60 to 75% of pregnant women have gingivitis, an early stage of periodontal disease that occurs when the gums become red and swollen from inflammation that may be aggravated by changing hormones during pregnancy [24]. If gingivitis is not treated, periodontitis occurs. Periodontitis has also been associated with poor pregnancy outcomes, including pre-term birth and low birth weight [25], although how periodontitis may lead to adverse pregnancy outcomes is not yet fully understood. Pregnant women may also be at risk of cavities due to changes in behaviors such as eating habits [26]. In addition, it has also been shown that women who have a lot of cavity-causing bacteria during pregnancy and after delivery could transmit these bacteria from their mouth to the mouth of their baby [27]. 

In this scenario, ozonated water appears as a biocompatible agent with a good antiseptic and antimicrobial potential role, potentially being useful for periodontal therapy. Ozone has been recently advocated predominantly due to its antimicrobial action which results from oxidation of microbial cellular components, altering the subgingival homeostasis. When ozone dissolves in water, hydroxyl radicals are generated, which are highly unstable. Thus, the antimicrobial action is caused by direct reactions of molecular ozone and other free-radical-mediated reactions.

In the present study, the improvement observed in gingival inflammation may have been due to a reduction in inflammation that may be attributed to the antimicrobial properties of ozone, also considering that no toothpaste with active ingredients that can modulate the inflammatory response, such as hyaluronium acid or lactoferrin, was prescribed [28]. An improvement in gingival status after using ozonated water as a gingival irrigator was previously observed by other researchers, such as Dodwad et al. [29], Kshitish et al. [30], and Isaac et al. [31].

From these analyses, it can be observed how health care professionals such as dental hygienists have a fundamental role in promoting oral health in special-needs patients, such as pregnant women, to stimulate and motivate them in relation to the importance of oral health, and in promoting correct attitudes and a healthy lifestyle. In a recent survey of 385 pregnant women, gingival bleeding was the main symptom reported, followed by gingival redness and edema [32], and it was concluded that although pregnant women seem to be moderately informed about the importance of oral health during pregnancy, health care professionals do not seem to participate actively, as they need to inform pregnant patients more actively about the importance of preventive oral health measures and oral health care during pregnancy. In another recent survey, it was found that most dentists agreed about the timing of conducting various dental procedures, about the administration of anesthetics and other drugs during pregnancy, and that dental care should be part of prenatal care [33]. However, uncertainty was observed regarding the relationship between periodontal disease and adverse pregnancy outcomes, and about possible recommendations to give to pregnant women to preserve their gingival health. In this effort, an ozonated water irrigator could be considered a valid device to manage home oral hygiene in pregnant women, also considering the unclear results with the use of chlorhexidine regarding the small risk, or lack of difference in risk, of caries presence in primary teeth between antimicrobial (chlorhexidine) and placebo treatment in mothers’ dentition (according to the results from three clinical trials involving 479 participants with low-certainty evidence) [34]. 

In the present study, the patients belonging to the study group started at T0 with a lower PI than the patients belonging to the control group, who started with a higher PI. This finding suggests that the subjects in the control group had poor oral cleaning abilities at the beginning of the period. However, the repeated measures analysis of variance found that the PI values systematically decreased over time in both groups, confirming the fundamental role of promoting oral hygiene in special-needs patients, such as pregnant women, to stimulate and motivate them in relation to the importance of oral health. 

## 5. Conclusions

From the results obtained on the PI, pregnant women who were re-motivated and educated on correct home oral hygiene by health care professionals showed an improvement in their PI over time. From the results obtained on the BOP index, it seemed that the pregnant women in the study group compared to the women in the control group had a decrease in the BOP index over time, due to the beneficial properties of ozonated water.

## Figures and Tables

**Table 1:** Plaque index in the two groups over time.

	T0			T1			T2		
	Mean ± Std Error	95% Confidence Interval		Mean ± Std Error	95% Confidence Interval		Mean ± Std Error	95% Confidence Interval	
		Lower Bound	Upper Bound		Lower Bound	Upper Bound		Lower Bound	Upper Bound
Study group	32.43 ± 6.01 ϕ	19.85	45.01	6.00 ± 1.54 ϕ	2.77	9.23	3.93 ± 1.70 ϕ	0.38	7.48
Control group	44.57 ± 8.5 ϕ,*	26.79	62.36	18.43 ± 2.18 ϕ,*	13.86	22.1	15.71 ± 2.18 ϕ,*	13.86	20.73

* Between-group differences (*p* < 0.05); ϕ intra-group significant differences.

**Table 2:** Bleeding on probing index (BOP) in the two groups over time.

	Mean ± SD	Mean ± SD	Mean ± SD
Study group	12 ± 10.13	4.07 ± 3.20	3.0 ± 3.06
Control group	9.29 ± 11.38	6.57 ± 3.36	8.57 ± 10.34

## Data Availability

The data that support the findings of this study are available from the University Vita-Salute San Raffaele, but restrictions apply to the availability of these data, which were used under license for the current study and thus are not publicly available. Data are, however, available from the authors upon reasonable request.

## References

[1] Neuman H., Koren O. (2017). The Pregnancy Microbiome. Intestinal Microbiome: Functional Aspects in Health and Disease.

[2] Yang I., Claussen H., Arthur R.A., Hertzberg V.S., Geurs N., Corwin E.J., Dunlop A.L. (2022). Subgingival Microbiome in Pregnancy and a Potential Relationship to Early Term Birth. Front. Cell. Infect. Microbiol..

[3] Zakaria Z.Z., Al-Rumaihi S., Al-Absi R.S., Farah H., Elamin M., Nader R., Bouabidi S., Suleiman S.E., Nasr S., Al-Asmakh M. (2022). Physiological Changes and Interactions Between Microbiome and the Host During Pregnancy. Front. Cell. Infect. Microbiol..

[4] Figueiredo C.S.D.A., Rosalem C.G.C., Cantanhede A.L.C., Thomaz B.A.F., Da Cruz M.C.F.N. (2017). Systemic alterations and their oral manifestations in pregnant women. J. Obstet. Gynaecol. Res..

[5] Butera A., Maiorani C., Morandini A., Simonini M., Colnaghi A., Morittu S., Barbieri S., Ricci M., Guerrisi G., Piloni D. (2021). Assessment of Oral Microbiome Changes in Healthy and COVID-19-Affected Pregnant Women: A Narrative Review. Microorganisms.

[6] La X., Jiang H., Chen A., Zheng H., Shen L., Chen W., Yang F., Zhang L., Cai X., Mao H. (2022). Profile of the oral microbiota from preconception to the third trimester of pregnancy and its association with oral hygiene practices. J. Oral Microbiol..

[7] Jeihooni A.K., Jamshidi H., Kashfi S.M., Avand A., Khiyali Z. (2017). The Effect of Health Education Program Based on Health Belief Model on Oral Health Behaviors in Pregnant Women of Fasa City, Fars Province, South of Iran. J. Int. Soc. Prev. Community Dent..

[8] Marchetti E., Casalena F., Capestro A., Tecco S., Mattei A., Marzo G. (2015). Efficacy of two mouthwashes on 3-day supragingival plaque regrowth: A randomized crossover clinical trial. Int. J. Dent. Hyg..

[9] Marchetti E., Tecco S., Caterini E., Casalena F., Quinzi V., Mattei A., Marzo G. (2017). Alcohol-free essential oils containing mouthrinse efficacy on three-day supragingival plaque regrowth: A randomized crossover clinical trial. Trials.

[10] Gallo S., Scribante A. (2021). Ozone therapy in dentistry: From traditional applications towards innovative ones. A review of the literature. IOP Conf. Ser. Earth Environ. Sci..

[11] Butera A., Scribante A., Maiorani C., Chiesa A., Lanteri V., Esposito F., Segù M., Baena R.R.Y. (2021). Split mouth randomized controlled trial: Standard therapy vs ultrasonic therapy with ozone gas application. Int. J. Clin. Dent..

[12] Colombo M., Gallo S., Garofoli A., Poggio C., Arciola C., Scribante A. (2021). Ozone Gel in Chronic Periodontal Disease: A Randomized Clinical Trial on the Anti-Inflammatory Effects of Ozone Application. Biology.

[13] Monzillo V., Lallitto F., Russo A., Poggio C., Scribante A., Arciola C.R., Bertuccio F.R., Colombo M. (2020). Ozonized Gel Against Four Candida Species: A Pilot Study and Clinical Perspectives. Materials.

[14] Lauritano D., Carinci F., Palmieri A., Girardi A., Cura F. (2015). Aquolab^®^ ozone-therapy is an efficient adjuvant in the treatment of chronic periodontitis: A case-control study. J. Orofac. Sci..

[15] Scribante A., Gallo S., Pascadopoli M., Soleo R., Di Fonso F., Politi L., Venugopal A., Marya A., Butera A. (2022). Management of Periodontal Disease with Adjunctive Therapy with Ozone and Photobiomodulation (PBM): A Randomized Clinical Trial. Photonics.

[16] Sridharan K., Sivaramakrishnan G. (2021). Interventions for oral lichen planus: A systematic review and network meta-analysis of randomized clinical trials. Aust. Dent. J..

[17] Küçük F., Yıldırım S., Çetiner S. (2021). Cytotoxicity assessment of different doses of ozonated water on dental pulp cells. BMC Oral Health.

[18] Shichiri-Negoro Y., Tsutsumi-Arai C., Arai Y., Satomura K., Arakawa S., Wakabayashi N. (2021). Ozone ultrafine bubble water inhibits the early formation of Candida albicans biofilms. PLoS ONE.

[19] Butera A., Gallo S., Pascadopoli M., Luraghi G., Scribante A. (2021). Ozonized Water Administration in Peri-Implant Mucositis Sites: A Randomized Clinical Trial. Appl. Sci..

[20] Meroni C. (2016). Valutazione della potenziale tossicità sull’uomo, per dispersione ambientale, di ozono proveniente dell’apparecchiatura per l’igiene orale Aquolab. Report of the President of the Council of the Order of Chemists.

[21] Jeffcoat M.K., Mcguire M., Newman M.G. (1997). Evidence-Based Periodontal Treatment Highlights from the 1996 World Workshop in Periodontics. J. Am. Dent. Assoc..

[22] Villa A., Abati S., Pileri P., Calabrese S., Capobianco G., Strohmenger L., Ottolenghi L., Cetin I. (2013). GG Campus Oral health and oral diseases in pregnancy: A multicentre survey of Italian postpartum women. Aust. Dent. J..

[23] Silla J.M.A., Pastor P.J.A., Catalá M.B., Arcís C.B., Montiel-Company J.M. (2017). Socioeconomic factors and severity of periodontal disease in adults (35–44 years). A cross sectional study. J. Clin. Exp. Dent..

[24] ADA Council on Access, Prevention and Interprofessional Relations, ADA Council on Scientific Affairs (2006). Using mouthguards to reduce the incidence and severity of sports-related oral injuries. J. Am. Dent. Assoc..

[25] Stefano C., Silvio T., Massimo D.F., Luca F., Roberto W., Enrico F. (2016). Adverse pregnancy outcomes and periodontitis: A systematic review and meta-analysis exploring potential association. Quintessence Int..

[26] Azofeifa A., Yeung L.F., Alverson C.J., Beltrán-Aguilar E. (2016). Dental caries and periodontal disease among U.S. pregnant women and nonpregnant women of reproductive age, National Health and Nutrition Examination Survey, 1999–2004. J. Public Health Dent..

[27] Dye B.A., Vargas C.M., Lee J.J., Magder L., Tinanoff N. (2011). Assessing the Relationship Between Children’s Oral Health Status and That of Their Mothers. J. Am. Dent. Assoc..

[28] Scribante A., Butera A., Alovisi M. (2022). Customized Minimally Invasive Protocols for the Clinical and Microbiological Management of the Oral Microbiota. Microorganisms.

[29] Dodwad V., Gupta S., Kumar K., Sethi M., Masamatti S. (2011). Changing paradigm in pocket therapy. Int. J. Public Health Dent..

[30] Kshitish D., Laxman V.K. (2010). The use of ozonated water and 0.2% chlorhexidine in the treatment of periodontitis patients: A clinical and microbiologic study. Indian J. Dent. Res..

[31] Issac A.V. (2015). Management of Chronic Periodontitis Using Subgingival Irrigation of Ozonized Water: A Clinical and Microbiological Study. J. Clin. Diagn. Res..

[32] Lazaridi I., Zekeridou A., Schaub L., Prudente D., Razban M., Giannopoulou C. (2022). A Survey on Oral Health Knowledge, Attitudes and Practices of Pregnant Women Attending Four General Health Hospitals in Switzerland. Oral Health Prev Dent..

[33] Razban M., Giannopoulou C. (2020). Knowledge and Practices of Oral Health Care During Pregnancy: A Survey Among Swiss Dentists. Oral Health Prev Dent..

[34] Riggs E., Kilpatrick N., Slack-Smith L., Chadwick B., Yelland J., Muthu M.S., Gomersall J.C. (2019). Interventions with pregnant women, new mothers and other primary caregivers for preventing early childhood caries. Cochrane Database Syst. Rev..

